# Autologous and Pooled Tumor Lysates in Combined Immunotherapy of Patients with Glioblastoma

**DOI:** 10.17691/stm2020.12.2.04

**Published:** 2020

**Authors:** S.V. Mishinov, A.Ya. Budnik, V.V. Stupak, O.Yu. Leplina, T.V. Tyrinova, A.A. Ostanin, E.R. Chernykh

**Affiliations:** Senior Researcher, Novosibirsk Scientific Research Institute of Traumatology and Orthopedics named after Ya.L. Tsivyan of the Ministry of Health of the Russian Federation, 17 Frunze St., Novosibirsk, 630091, Russia;; Resident, Novosibirsk Scientific Research Institute of Traumatology and Orthopedics named after Ya.L. Tsivyan of the Ministry of Health of the Russian Federation, 17 Frunze St., Novosibirsk, 630091, Russia;; Professor, Head of Neurosurgery Research Department, Novosibirsk Scientific Research Institute of Traumatology and Orthopedics named after Ya.L. Tsivyan of the Ministry of Health of the Russian Federation, 17 Frunze St., Novosibirsk, 630091, Russia;; Leading Researcher, Laboratory of Cellular Immunotherapy, Scientific Research Institute of Fundamental and Clinical Immunology, 14 Yadrintsevskaya St., Novosibirsk, 630099, Russia;; Researcher, Laboratory of Cellular Immunotherapy, Scientific Research Institute of Fundamental and Clinical Immunology, 14 Yadrintsevskaya St., Novosibirsk, 630099, Russia;; Professor, Chief Researcher, Laboratory of Cellular Immunotherapy, Scientific Research Institute of Fundamental and Clinical Immunology, 14 Yadrintsevskaya St., Novosibirsk, 630099, Russia;; Professor, Corresponding Member of the Russian Academy of Sciences, Head of the Laboratory of Cellular Immunotherapy, Scientific Research Institute of Fundamental and Clinical Immunology, 14 Yadrintsevskaya St., Novosibirsk, 630099, Russia

**Keywords:** glioblastoma, immunotherapy, dendritic cells, overall survival of glioblastoma patients, autologous tumor lysate, pooled tumor lysate

## Abstract

Although major progress has been made in the standard treatment for glioblastomas, encompassing the maximal surgical resection, chemotherapy and radiation therapy, it is possible to increase survival rates significantly only in a few patients. Therefore, it is necessary to explore new therapeutic modalities, one of which is immunotherapy.

**The aim of the study** was to evaluate the efficacy of the combined use of autologous and pooled tumor lysates in comprehensive treatment of patients with glioblastoma.

**Materials and Methods:**

All patients (n=58, including 30 males and 28 females aged 18–70 years) were randomized into three groups, two of which received immunotherapy based on injection of autologous dendritic cells pulsed with autologous tumor lysates (first protocol) or pooled lysates (second protocol) from more than one tumor, in addition to the planned standard treatment. The patients of group 3 (control) received the standard comprehensive treatment encompassing the maximum safe tumor resection followed by radiation therapy and chemotherapy.

**Results:**

The tolerability of both applied immunotherapy protocols was good: there were no anaphylactic reactions observed or patients who prematurely discontinued participation in the study. The final analysis of the data revealed no significant differences in median survival values of patients in each of the three groups. However, when analyzing the Karnofsky Performance Status in patients of group 2, it was found that it tended to improve.

**Conclusion:**

The study shows that the proposed immunotherapy protocols are safe for clinical use and have the potential to improve the patient’s life quality. However, these findings should be considered intermediate until the findings of multicenter randomized clinical trials with a larger number of patients are obtained.

## Introduction

Glioblastoma is a primary malignant brain neoplasm most common in the adult population [1–4], though it can be detected in patients of any age [4]. This tumor accounts for approximately 15% of all primary brain tumors and about 45–50% of all primary malignant neoplasms of the brain [4]. In European countries, North America and Australia, the incidence of glioblastoma is 3 to 10 cases per 100,000 population/year [1, 5]. In the literature, we have not found the exact incidence rates for glioblastoma in Russia, which is probably due to the lack of the unified register of such tumors [6]. Glioblastoma is a fatal disease causing most patients to die within 15 months after diagnosis [7]. It mostly affects people of the older age group, the peak incidence occurring in patients aged between 55 and 85 years [4].

Over the past decades, certain advances have been achieved in the management of glioblastoma. However, even the combination of maximum surgical resection, radiotherapy, and chemotherapy with temozolomide, currently applied as the standard therapy, made it possible to achieve only a five-percent average five-year survival due to the high degree of glioblastoma resistance to these therapeutic modalities, among other reasons [8, 9].

Recurrence occurs in the vast majority of patients. Resistance to therapy is attributable to various factors [4, 9]:

1. the presence of a partially preserved blood-brain barrier in the tumor tissue prevents the pass of antitumor drugs into it;
2. invasive properties of glioblastoma cells enable them to spread over large distances within the central nervous system and remain enclosed by an intact blood-brain barrier;
3. the heterogeneity of tumor cells and their genomic instability lead to the emergence of clonal populations of resistant cells, therefore making it necessary to monitor these processes;
4. the presence of a population of tumor-initiating or stem-like cells can serve as a resistance reservoir;
5. tumor progression can induce secondary oncogenic changes.

It is also well known that glioblastoma cells are able to avoid the host immune response through expression of various immunosuppressive factors and induction of effector T lymphocyte apoptosis [10]. These factors include indolamine 2,3-dioxygenase (IDO), transforming growth factor β (TGF-β), as well as signal transducer and activator of transcription 3 (STAT3). In particular, IDO catalyzes the conversion of tryptophan to kynurenine, which in turn triggers apoptosis of effector T cells and immunosuppression mediated by regulatory T cells (Treg). Glioma cells also produce chemokine CCL2, which serves as an attractant of regulatory T cells [11]. Treg cells are a subset of CD4^+^ T lymphocytes expressing the FoxP3 transcription factor, CD25 receptor (CD25^+^) with high affinity for IL-2, and cytotoxic T lymphocyte antigen 4 (CTLA-4). CTLA-4 effects are mainly observed in naive and resting T lymphocytes and mediate suppression of T killers, complementing the inhibitory activity of Treg cells responsible for maintaining immune tolerance throughout life [10–14]. Treg cells accumulate in gliomas and the perifocal zone during the entire tumor progression, while the intensity of infiltration correlates with the degree of tumor malignancy [15, 16]. Treg cells were found to express B7-H1, a synonym for programmed cell death receptor ligand (PD-L1), on their surface. This transmembrane protein is a negative co-stimulatory molecule acting as an inhibitor of proliferation and inducer of antigen-specific CD8^+^ T cell death. Treg cells play an important role in suppressing the immune response by gliomas as confirmed by a number of studies [17–19].

Tumor cells were found in the blood of some patients with glioblastoma [20]. However, it is well known that this tumor extremely rarely forms metastases beyond the central nervous system. This fact along with the evidence that this tumor also developed in the organs transplanted to recipients from donors with glioblastoma during immunosuppressive therapy [21] suggests that the immune system normally suppresses the metastatic potential of glioblastoma cells circulating in the blood.

Given the low susceptibility of this neoplasm to standard therapeutic and surgical interventions and high mortality, the scientific search for alternative treatment methods is increasingly relevant. In the last decade, several potentially promising methods have been developed, in particular, targeted therapy and immunotherapy [7].

The immunotherapeutic methods for tumor treatment include the use of tumor-specific T cell vaccines, injections of sensitized dendritic cells, peptide vaccines, as well as immunovirotherapy [22–24]. There are relatively few completed clinical trials of immunotherapy for the treatment of patients with glioblastoma. Nevertheless, data on this issue are gradually accumulating. For example, Fadul et al. [25] showed the presence of immune response and increased survival in patients who received injections of autologous sensitized dendritic cells in a small cohort study back in 2011. There are findings of more recent works that indicate the promising potential of immunotherapeutic methods in the treatment of such patients [26, 27].

**The aim of the study** was to evaluate the efficacy of the combined use of autologous and pooled tumor lysates in comprehensive treatment of patients with glioblastoma.

The following tasks were solved:

comparative assessment of tolerability and safety of combined immunotherapy (CIT) according to two different protocols in the comprehensive treatment of patients with histologically verified supratentorial glioblastoma;

comparison of survival curves of patients in groups with and without the use of CIT, as well as in groups with immunotherapy where the first or second CIT protocols were used.

## Materials and Methods

The study involved 58 patients of Neurosurgery Department of Novosibirsk Scientific Research Institute of Traumatology and Orthopedics named after Ya.L. Tsivyan, including 30 males and 28 females. Inclusion criteria were age 18 to 70 years, morphologically verified diagnosis of glioblastoma (Grade IV), the maximum safe tumor resection, at least 60 points on Karnofsky Performance Status (KPS) at the initiation of CIT, absence of other oncological diseases, absence of immunodeficiency conditions, absence of severe and/or decompensated concomitant pathology. Regardless of the CIT stage, exclusion criteria were manifestations of anaphylactic reaction, a patient’s refusal of therapy.

An individual electronic case report form was completed for each patient; therapy was started after signing an informed consent to participate in a pilot study.

The study complies with ethical principles established by the Declaration of Helsinki (2013) and the Rules of Good Clinical Practice provided in the Order of the Ministry of Health the Russian Federation No.200n dated April 1, 2016. The study was performed following approval by the Biomedical Ethics Committee of Scientific Research Institute of Traumatology and Orthopedics named after Ya.L. Tsivyan, the Ethics Committee of Scientific Research Institute of Fundamental and Clinical Immunology.

CIT tolerability was evaluated using the developed scale during the entire treatment period once a week during a visit to the specialist. The results of all visits were reviewed with a score characterizing the presence of adverse events. The score of four points corresponded to good tolerability of therapy. One point was subtracted from the initial four points for the presence of each symptom indicated in Table 1. Anaphylactic reactions (anaphylactic shock, Quincke’s edema) were not included in the evaluation criteria, since they were automatically the signs of treatment intolerance and an unsatisfactory mark on the proposed scale. If they developed, the therapy was stopped, the patients were excluded from the study. A score of three points corresponded to a satisfactory mark, two points or less implied unsatisfactory CIT tolerability.

**Table 1:** Overall assessment of combined immunotherapy tolerability

Score	Signs of intolerance
–1 point	Body temperature above 38°C for more than 24 h from initiationof combined immunotherapy
–1 point	Local and systemic skin reactions (rash, hyperemia) and/or local and systemic itching
–1 point	Presence of “new” complaints (nausea, weakness, dizziness, headaches) associated with combined immunotherapy

Safety was assessed using laboratory tests: general blood and urine tests, biochemical blood tests.

Two different CIT protocols were analyzed in the study [28]: the first was based on the use of a patient’s own (autologous) tumor lysates, and the second was based on the use of pooled (allogeneic) human glioblastoma lysates.

*The first protocol* was based on specific antitumor immune response activation. Immunotherapy using dendritic cell vaccines was carried out in accordance with the developed patent [29].

The dendritic cells of patients were obtained *ex vivo* from peripheral blood monocytes. For this purpose, Ficoll-Verographin density gradient was used to isolate a fraction of mononuclear cells from 250–300 ml of peripheral blood taken the day before surgery. This fraction was enriched with monocyte content using two-hour adhesion to plastic in RPMI-1640 medium (Sigma-Aldrich, USA) supplemented with 0.3 mg/ml L-glutamine, 5 mM HEPES buffer, 100 μg/ml gentamicin and 2.5% fetal bovine serum (FCS; BioloT, Russia). Next, adherent cells were incubated for 3 days in the complete culture medium in presence of granulocyte macrophage colony-stimulating factor (GM-CSF, 40 ng/ml; Sigma-Aldrich) and interferon-alpha (IFN-α, 1000 Units/ml, Roferon-A; Roche, Switzerland) at 37°C and 5% CO_2_. Maturation of dendritic cells (DC) was induced by adding azoximer bromide (Polyoxidonium; Petrovax Pharm, Russia) at a dose of 2 ng/ml for 24 h followed by treatment with autologous tumor cell lysate at a dose of 5 mg/ml protein for 1 h.

To obtain lysate, fragments of tumor removed from patients during neurosurgical intervention were subjected to 5 cycles of freezing/thawing. After the resulting suspension was centrifuged, the supernatant was taken and the concentration of the released tumor antigens (mg/ml protein) was measured in it. Tumor lysates of patients (as a complete set of tumor antigens) were stored at –20°C.

The antigen-loaded DC obtained this way were preserved in a cryo-solution containing 10% dimethyl sulfoxide — DMSO (Sigma-Aldrich, USA) and 90% human albumin solution (Microgen, Russia), and stored at –80°C up to the time of vaccination procedures. As it was planned, DC vaccination was performed in the form of 4–6 subcutaneous injections at the average dose of 5·10^6^ cells once in 2 weeks in combination with chemotherapy after standard radiation therapy. However, it remained possible even in cases when patients did not undergo a complete course of standard treatment, since vaccines were already prepared for them at hospitalization and surgical treatment stage. Recombinant IL-2 (Roncoleukin; Biotech, Russia) was used as adjuvant and was administered subcutaneously at a dose of 250,000 units near the injection site.

*The second protocol* was also aimed at activating specific antitumor immune response, but there was a difference. According to the second protocol, 250–300 ml of peripheral blood for generating DC was to be collected not the day before surgery, but at the stage prior to chemotherapy. If a patient did not receive a complete course of standard comprehensive treatment for some reason, they still remained in the group and were administered vaccination as well, because, according to the design, incomplete course of comprehensive treatment was not a criterion for exclusion from the study.

The second protocol had another significant difference: maturation of DC was carried out using a load of lysate (5 mg/ml protein) of the pooled tumor antigens obtained from various patients during previous neurosurgical interventions. The lysate was obtained according to the method described above in the first protocol. After a one-hour loading with tumor antigens, azoximer bromide (2 ng/ml) was added to three-day-old cultures of immature DC for 24 h. Cryopreservation and storage of DC obtained in this way were carried out according to the standard procedure.

Immunotherapy of the modified protocol involved two vaccination courses. The first course consisted of 4–6 subcutaneous injections of DC loaded with pooled tumor antigens into the interscapular region (the average dose of 5·10^6^ cells) with a two-week interval (total duration 3 months). The second course was carried out after completing the first one. It consisted of 4–6 vaccinations given once a month during 6 months. Recombinant IL-2 (Roncoleukin) was used as adjuvant. Vaccinations were performed subcutaneously at 4 points in the interscapular region, IL-2 was administered in the similar manner at 4 points near the injection site.

All patients were divided into three groups: groups 1 (n=18) and 2 (n=9) received CIT according to the first and second protocol, respectively. The treatment was carried out in addition to the planned standard radiotherapy and chemotherapy. In group 3 (n=31), patients underwent surgical treatment followed by standard comprehensive treatment without CIT.

Since the beginning of 2014, all patients under study have been operated at the Neurosurgical Department No.1 of Novosibirsk Scientific Research Institute of Traumatology and Orthopedics named after Ya.L. Tsivyan using identical surgical techniques. The maximum safe tumor resection was performed under optical magnification with OPMI Vario 33 microscope (Carl Zeiss, Germany) using intraoperative navigation and neurophysiological monitoring (if indicated). In all cases, surgical material was collected for histological examination. In patients recruited to group 1, tumor material at least 2 cm^3^ in volume was also taken for utilization as an autologous antigen for generating DC in the framework of the first CIT protocol. The collected material was placed in an airtight container filled with physiological saline and transported immediately to the immunology laboratory. In the first 48 h after surgery, all patients underwent control MRI scan to assess the extent of tumor resection objectively.

According to the developed design, introduction of DC vaccines was planned by the time after completing the course of radiation therapy due to possible presence of immunosuppression and the use of synthetic glucocorticoids. However, in cases when patients did not receive a complete course of standard comprehensive treatment, they were not excluded from the study. They received CIT depending on the protocol in which these patients were recruited initially.

At the stage of surgical treatment, synthetic glucocorticoids (dexamethasone) were prescribed only symptomatically if required, the drug dose ranging from 4 to 24 mg/day. Thus, in all three groups there were some patients who did not receive the above drug. At the stage of radiation therapy, all patients were prescribed dexamethasone tablets at an average dose of 4 mg/day.

**Statistical data processing.** Patients were randomized at the final stage of forming the study design by generating random numbers in Random Number Generator 1.4 software. The distribution of patients in study groups is given in Table 2. The results were analyzed using the Statistica v. 10.0 software (Statsoft Inc., USA).

**Table 2:** Distribution of patients in study groups

Parameters	Group 1	Group 2	Group 3 (without combined immunotherapy)
Total number of patients:	18		31
males	10		18
females	8		13
Mean age (years)	53.3	52	56.2
Karnofsky Performance Status at the beginning of combined immunotherapy (points), Me [25; 75]	60 [50; 70]	70 [70; 80]	60 [40; 80]

Given the small population sampling of the study, discrete variables were analyzed using the nonparametric Wilcoxon–Mann–Whitney rank-based method. Patient survival was assessed using Kaplan–Meier curves. Frequency variables were analyzed using χ^2^ method. The alpha level for deciding whether to accept or reject the null hypothesis was assumed to be 0.05.

Age values were presented as the mean, KPS values were presented as medians (Me), as well as the first and third quartiles [Q1; Q3].

## Results

**Figure 1:**
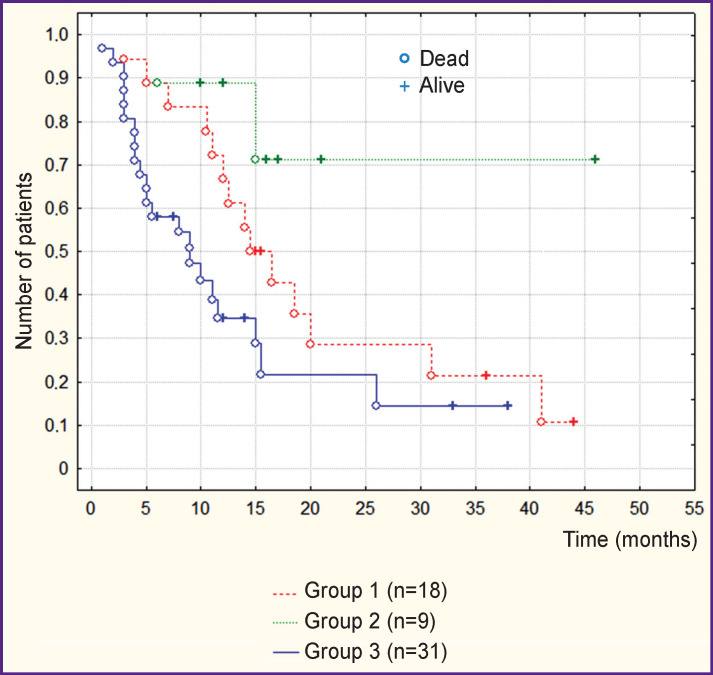
Curves of overall survival in the initially recruited groups of patients

Analysis of the overall survival curves showed statistically significant differences in the median indices between groups 1 and 3 (p<0.05) and groups 2 and 3 (p<0.05) (Figure 1). There were no statistically significant differences between groups 1 and 2 (p>0.05). At the same time, it was found that 55% of patients of group 3 (without immunotherapy) did not receive a complete course of standard comprehensive treatment (radiation therapy and/or chemotherapy), while patients with recurrence prevailed in groups 1 and 2: 72 and 67%, respectively. Given this fact, it seemed incorrect to rely on the overall survival curves to draw conclusions, since the statistically significant difference in median survival values could be attributable to these factors. Therefore, survival curves were evaluated only in that proportion of patients who received comprehensive treatment (radiation therapy and chemotherapy) in full measure. The groups formed according to the new principle (n_1_=12, n_2_=7, n_3_=10) had no differences in gender, age, or KPS. Besides, patients in these groups received similar average course doses of glucocorticoids both at the stage of surgical treatment and at the stage of radiation therapy.

**Figure 2:**
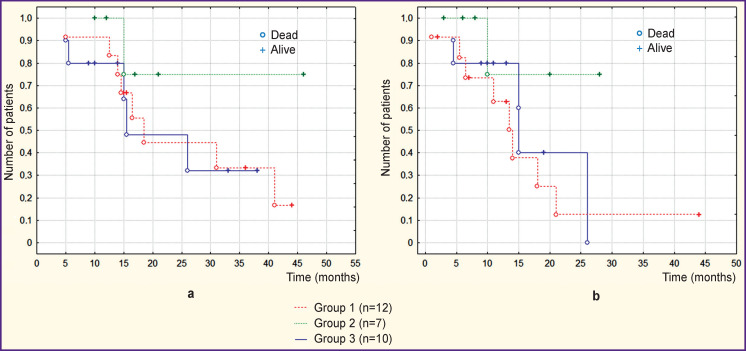
Survival in the newly formed groups of patients who received standard comprehensive treatment: (a) overall survival curves; (b) curves of survival since the last operation performed

Patients with recurrent disease still prevailed in groups with immunotherapy: 10 out of 12 in group 1, and 5 out of 7 in group 2, while in group 3 there were only 2 patients with recurrence out of 10. These indicators were significantly different between groups 1 and 3 (p<0.05) in contrast to groups 2 and 3 where the differences were not significant (p>0.05). The comparative analysis demonstrated no statistically significant differences between the groups in the values of either median overall survival or median survival since the last performed operation (Figure 2). In our series, this suggested that the fact of recurrence did not affect survival. Median survival values in groups are given in Table 3.

**Table 3:** Median survival rates in the newly formed groups of patients who received standard comprehensive treatment

Groups	Median overall survival (months)	Median survival since the last operation (months)
Group 1 with autologous lysate (n=12)	16	12
Group 2 with pooled lysate (n=7)	15	10
Group 3 without immunotherapy (n=10)	14.5	12

It was revealed during analysis of patients’ KPS that in the long-term period (more than 6 months after the first surgery), this value demonstrated a trend to improve by 10 points in patients from group 1. Me_KPS_ was 60 [50; 70] points (see Table 2) at the beginning of CIT, while it equaled 70 [60; 80] points at the time of screening in living patients. A similar change was observed in patients of group 2, though it was not significant. In the long-term period, KPS remained unchanged among the survived patients of group 3.

It was revealed in the study that treatment tolerability was good in both CIT protocols, the median corresponded to 4 points. There were no premature patient withdrawals or anaphylactic reactions.

## Discussion

What deserves particular attention in the present study is heterogeneity of patients in the formed groups. Randomization took place before initiation of surgical treatment, therefore the researchers had no idea about the exact extent of appropriate standard treatment a certain patient would receive. In general, the majority of patients — 27.6% (16 out of 58) — did not receive radiation therapy or chemotherapy (i.e. only surgical treatment was performed). Lower KPS was probably due to the following circumstances: patients who did not receive comprehensive treatment had Me_KPS_=60 [50; 70] points and 50 [40; 60] points after surgery, while the rest of the patients had Me_KPS_ =70 [60; 80] points both before and after surgery.

As the study shows, KPS is not a fundamental factor for refusing to administer chemotherapy to a patient, since patients usually receive treatment parenterally at home. In case of satisfactory somatic status, chemotherapy should be prescribed regardless of neurological deficit, which is often associated with low KPS. Rejection of this fact may become a significant drawback of cancer management system and serve as a significant negative factor in evaluating the efficacy of the proposed new adjuvant methods. In our study, the comparison group has been reduced by three times due to heterogeneity of standard comprehensive treatment, which reconfirms the complexity of clinical trials in patients with glioblastoma.

After analyzing the results of treatment according to the proposed protocols [28], we came to the conclusion that the first immunotherapy protocol, despite its good tolerability, had a number of disadvantages:

difficulty of predicting the completeness of subsequent comprehensive treatment of patients (radiation therapy and chemotherapy) as the patient is included in the protocol before surgery, the vaccine is made while the patient is in the neurosurgical department, and then it is kept frozen until the patient receives the appropriate radiation therapy; if the patient’s KPS decreases after surgery, they are usually refused radiation therapy, though the vaccine has already been made for them;

heterogeneity of antigenic (tumor) material obtained during surgery;

presence of concomitant procedures potentially influencing the disease course as a whole (hemoexfusion to isolate immune cells, taking glucocorticoids).

It is necessary to note that in the first protocol, hemoexfusion for generating dendritic cells was performed in the preoperative period when a larger proportion of patients received glucocorticoids. The use of an autologous antigen dictated the conditions for creating a vaccine during the period corresponding to the patient staying in the neurosurgical hospital. Technically, the first protocol was more difficult to perform for a number of reasons. Surgical interventions were based on the timing of immunocompetent cell generation cycles and therefore performed on a specific day; intraoperative sampling of tumor antigenic material excluded patients operated on at other clinical bases and patients with relapse who did not undergo surgery due to contraindications or an insignificant tumor volume. In this regard, the second protocol was more flexible, not tied to surgical intervention and autologous antigen harvesting.

Thus, evaluation of glioblastoma treatment outcomes in patients managed according to the proposed protocols showed no significant differences in their survival rates, the tolerability of immunotherapy was good in both cases. However, the combination of factors mentioned above allows us to conclude that the second protocol based on the use of pooled lysates has a number of advantages in practical application and can be recommended for multicenter clinical trials. To reduce heterogeneity in groups of patients while planning the studies on the efficacy of new therapeutic methods, it is important to consider the inclusion/exclusion criteria carefully. It is appropriate to exclude patients not receiving complete standard comprehensive treatment from the study groups.

## Conclusion

Combined immunotherapy protocols based on the use of dendritic cell vaccines are a safe adjuvant method in comprehensive treatment of patients with supratentorial gliomas and can potentially improve their Karnofsky Performance Status in the long term. Combined immunotherapy protocol with the use of pooled lysates has several advantages over the use of autologous lysates and can be recommended for clinical trials designed to evaluate the efficacy.
